# Glycosylated fibronectin as a first trimester marker for gestational diabetes

**DOI:** 10.1007/s00404-020-05670-8

**Published:** 2020-07-11

**Authors:** Julia Alanen, Heidi Appelblom, Teemu Korpimaki, Heikki Kouru, Mikko Sairanen, Mika Gissler, Markku Ryynanen, Jaana Nevalainen

**Affiliations:** 1 Department of Obstetrics and Gynecology, Medical Research Center, PEDEGO Research Unit, University of Oulu and Oulu University Hospital, PL 24, 90100 OYS Oulu, Finland; 2 PerkinElmer, Turku, Finland; 3 National Institute for Health and Welfare, Helsinki, Finland

**Keywords:** Gestational diabetes, First trimester screening, Biomarker, Fibronectin

## Abstract

**Purpose:**

To evaluate the performance of first trimester maternal serum glycosylated (*Sambucus nigra* lectin-reactive) fibronectin in prediction of gestational diabetes mellitus (GDM).

**Methods:**

In this case–control study, first trimester maternal serum glycosylated fibronectin and fibronectin were measured in 19 women who consequently developed GDM and in 59 control women with normal pregnancy outcomes. Adiponectin was used as a reference protein to evaluate relation of glycoprotein to SNA-lectin-reactive assay format. Samples were taken during gestational weeks 9^+6^–11^+6^. Data concerning GDM was obtained from the National Institute for Health and Welfare, which records the pregnancy outcomes of all women in Finland.

**Results:**

There was no difference in maternal serum glycosylated fibronectin concentrations between women with consequent GDM [447.5 μg/mL, interquartile range (IQR) 254.4–540.9 μg/mL] and control women (437.6 μg/mL, IQR 357.1–569.1 μg/mL). Maternal serum fibronectin levels were significantly lower in GDM group (224.2 μg/mL, IQR 156.8–270.6 μg/mL), compared to the control group (264.8 μg/mL, IQR 224.6–330.6 μg/mL, *p* < 0.01). There was no difference in assay formats for adiponectin.

**Conclusion:**

There was no association between first trimester maternal serum glycosylated (SNA-reactive) fibronectin and GDM.

## Introduction

Gestational diabetes mellitus (GDM) is a growing risk for both maternal and fetal health as its prevalence is increasing worldwide. In Finland, the prevalence of GDM was 15.6% in 2017 and 14.1% of women were obese [body mass index (BMI) ≥ 30 kg/m^2^] before pregnancy [1]. Increasing prevalence is worrisome as the adverse effects of GDM are not confined to pregnancy since it can also affect the later health of both mothers and children [2, 3]. Healthy lifestyle changes in diet and exercise can reduce the risk of GDM and the effect is stronger the earlier the intervention is made [4]. Currently, however, in most cases GDM is diagnosed with an abnormal oral glucose tolerance test (OGTT) only at gestational weeks 24–28. There is a need for an effective first trimester screening method for GDM to enable robust intervention.

Previous studies have shown that maternal serum biomarkers are altered already in the first trimester of pregnancy in women who subsequently develop GDM. Studied markers include metabolites such as glycine and arginine, fatty acids, sex hormone binding globulin (SHBG), inflammatory markers, adipocyte-derived markers, placenta-derived markers, placental exomes and glycosylated (*Sambucus nigra* lectin-reactive) fibronectin [5–12].

Some of the reports have been promising and marker combinations have been suggested to have up to 72% detection rate (DR) for a 20% false positive rate (FPR) [10]. However, published reports have been partly controversial and concerning maternal serum glycosylated fibronectin, that was reported to have excellent performance, there are only few published reports suggesting its usefulness as first trimester predictor of subsequent GDM [6, 13]. Fibronectin is a large dimeric glycoprotein that is expressed in various cell and tissue types and participates in multiple functions such as cell adhesion, growth, migration and differentiation. Cellular form of fibronectin is synthetized by endothelial cells, fibroblasts and smooth muscle cells. Fibronectin is one of the most reliable proteins that can be estimated as a plasma indicator protein for endothelial function and related pathological disorders [14–16]. Significant elevation of circulating fibronectin have been reported in various metabolic syndromes associated with endothelial function, such as diabetes [17–19]. The change in the levels of serum or plasma cellular fibronectin may reflect the extent of matrix changes and vessel wall damage in patients with diabetes. Although various forms of fibronectin have been implicated to have a role in metabolic disorders like diabetes the specific glycosylated version in question has only been investigated in just few studies [16]. Beside GDM, glycosylated plasma fibronectin has been indicated as an early predictor for pre-eclampsia, another major pregnancy disorder [20, 21].

Glycosylation of protein is an enzymatic process (e.g., various transferases), whereas related glycation process is a non-enzymatic process, where excess sugars are removed from body using so called amadori intermediate products, glycated hemoglobin (HbA1c) is one example of such protein.

Adiponectin is another glycoprotein that has been associated with GDM [22]. Adiponectin is a protein hormone that has a role in regulating glucose levels and fatty acid metabolism. In this study adiponectin was used as a comparator protein to evaluate SNA-lectin-based assay performance to direct sandwich immunoassay.

The aim of this study was to reevaluate the screening performance of first trimester maternal serum glycosylated fibronectin for GDM. For this purpose, the difference between first trimester glycosylated fibronectin values were compared between women with and without GDM.

## Materials and methods

The data for this retrospective case–control study was retrieved from first trimester combined screening program in Northern Finland during the study period of 1.1.2007–31.12.2011. Participation in combined screening program is voluntary. Maternal serum glycosylated fibronectin levels were measured from the frozen combined screening samples of a subset of women including 19 women who subsequently developed GDM and 59 control women who were not diagnosed with GDM. Small sample size was assumed to be sufficient based on high marker performance in original publication [16]. Power analysis assuming 80% sensitivity showed that with 20 cases the exact 95% confidence interval is 56.3–94.3%. As we did not aim to refute but to confirm previously reported performance level the small sample size was considered to be sufficient for this.

Women with multiple gestation or other pregnancy complications than GDM, were excluded from the study. There were no ethnic differences between the groups. Data concerning GDM was obtained from the National Institute for Health and Welfare, which records the outcome of all live births and stillbirths with a gestational age of 22^+°^ or more or a birth weight of 500 g or more in the country.

A 2-h 75 g OGTT during gestational weeks 24–28 or at 12–16 gestational weeks was used to diagnose GDM. Diagnostic cut-off values were ≥ 5.3 mmol/L (fasting blood glucose), ≥ 10.0 mmol/L (1 h) and ≥ 8.6 mmol/L (2 h). If one or more of the values were abnormal the woman was diagnosed as having GDM. Screening for GDM is universal in Finland but not all women undergo it since the OGTT is not recommended if the primipara is under 25 years with no family history of type 2 diabetes mellitus (T2DM) and with pre-pregnancy BMI of 18.5–25 or multipara is under 40 years with no previous GDM or macrosomia and with pre-pregnancy BMI under 25 kg/m^2^. OGTT during gestational weeks of 12–16 is recommended for women in increased risk of GDM (BMI ≥ 35, a previous GDM, glucosuria in the first trimester, polycystic ovary syndrome or a first-degree relative with T2DM). If early OGTT is negative, it is repeated at gestational weeks 24–28. Women with no testing for GDM were not included in this study.

We developed DELFIA research assays for fibronectin and glycosylated fibronectin. Normal fibronectin assay was done by coating 96-well plates with mouse monoclonal IgG antibody against human fibronectin (MAB1918, R&D Systems, Abingdon, UK). Sheep polyclonal Eu-N1-labeled Anti-hFibronectin antibody was used as a tracer (AF1918, R&D Systems, Abingdon, UK). To reduce unspecific binding in the assay for glycosylated (SNA-reactive) fibronectin the same primary coating antibody was pre-treated with Remove iT Endo S (New England BioLabs, Ipswich, MA, USA) reagent. Endo S is an endoglycosidase with a high specificity for removing N-linked glycans from the heavy chain of native IgG. Detection was done using biotinylated *Sambucus Nigra* Elderberry Bark Lectin (SNA) (Vector Laboratories, Burlingame, CA, USA) and Eu-SA (PerkinElmer, Waltham, MA, USA). Calibrators for both assays were made from human plasma derived fibronectin (1918-FN, R&D Systems, Abingdon, UK). Samples were diluted 1:2000 and 1:1000 for fibronectin and glycosylated (SNA-reactive) fibronectin assays, respectively. Run control coefficient of variation (CV) was 2.4% for both assays.

As a comparator for lectin-based assays we also developed DELFIA research assays for adiponectin and glycosylated adiponectin. Standard adiponectin assay was done by coating 96-well plates with mouse monoclonal IgG antibody against human adiponectin (MAB10651, R&D Systems, Abingdon, UK). Mouse monoclonal Eu-N1-labeled anti-hAdiponectin antibody was used as a tracer (MAB1065, R&D Systems, Abingdon, UK). To reduce unspecific binding in the assay for glycosylated (SNA-reactive) adiponectin the primary coating antibody was again pre-treated with Remove iT Endo S (New England BioLabs, Ipswich, MA, USA) reagent. Detection was done using biotinylated *Sambucus Nigra* Elderberry Bark Lectin (SNA) (Vector Laboratories, Burlingame, CA, USA) and Eu-SA (PerkinElmer, Waltham, MA, USA). Calibrators for both assays were made from recombinant human adiponectin (1065-AF, R&D Systems, Abingdon, UK). Samples were diluted 1:100 for adiponectin and glycosylated (SNA-reactive) adiponectin assays. Run control CV% was 4.0% for both assays.

Spotfire (TIBCO) was used to statistically analyze the results. Linear regression analysis was used to evaluate assay correlations. For continuous variables analysis of variance (ANOVA) and Chi square for categorical variables was used. Statistical significance was set for *p* value of < 0.05. Univariate logistic regression was done to create a ROC plot that was used to assess screening performance.

## Results

The mean age of women in the study group was 30.8 years (range 28.5–33.1) and 30.1 years (26.4–34.3) in the control group at sampling. There were no significant differences in gestational age at sampling, maternal weight or smoking habits. Maternal serum fibronectin levels were significantly lower (*p* = 0.007) in GDM group, 224.2 μg/mL (interquartile range (IQR) 156.8–270.6 μg/mL), compared to the control group 264.8 μg/mL (224.6–330.6 μg/mL, *p* < 0.01). There was, however, no statistical difference between the two groups in glycosylated fibronectin levels: 447.5 μg/mL (IQR 254.4–540.9 μg/mL) in the GDM group and 437.6 μg/mL (IQR 357.1–569.1 μg/mL) in the control group. Table 1 enlists maternal characteristics, OGTT results and median fibronectin and glycosylated fibronectin levels with IQRs in GDM and control group.

**Table 1:** Maternal characteristics and first trimester glycosylated fibronectin and fibronectin concentrations

	Nondiabetic controls (*n* = 59)	GDM (*n* = 19)	*p* value
Age (years)	30.1 (26.4–34.3)	30.8 (28.5–33.1)	0.65
Weight (kg)	67 (55–82)	77 (72–83)	0.09
Smoking (*n*, %)	9 (15%)	3 (16%)	0.95
GA* at sampling (days)	71 (70–81)	80 (71–83)	0.08
OGTT** (mmol/L)			
Fasting, 0 h	4.5 (3.9–4.7)	5.3 (5.0–5.6)	< 0.001
1 h	7.5 (6.5–9.1)	10.4 (9.1–11.3)	< 0.001
2 h	5.9 (5.4–6.7)	7.0 (6.3–8.4)	< 0.01
Fibronectin (μg/mL)	264.8 (224.6–330.6)	224.2 (156.8–270.6)	< 0.01
Glycosylated fibronectin (μg/mL)	437.6 (357.1–569.1)	447.5 (254.4–540.9)	0.35

Values are expressed as medians with interquartile range (IQR)

^*^GA gestational age

^**^OGTT oral glucose tolerance test

**Fig. 1:**
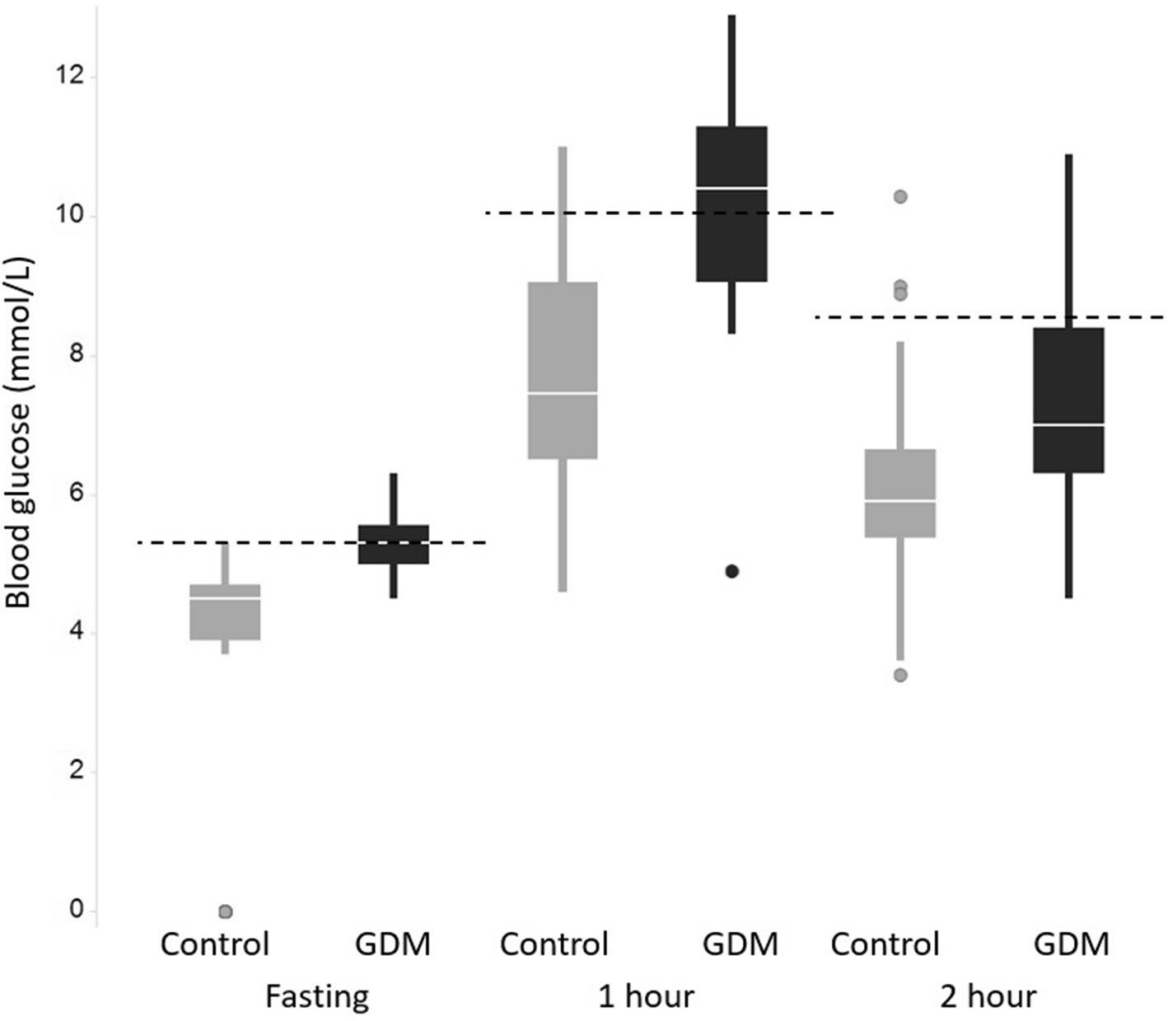
OGTT results for the control (*n* = 59) and GDM (*n* = 19) groups. Diagnostic threshold for each time point is marked with dottet-line

**Fig. 2:**
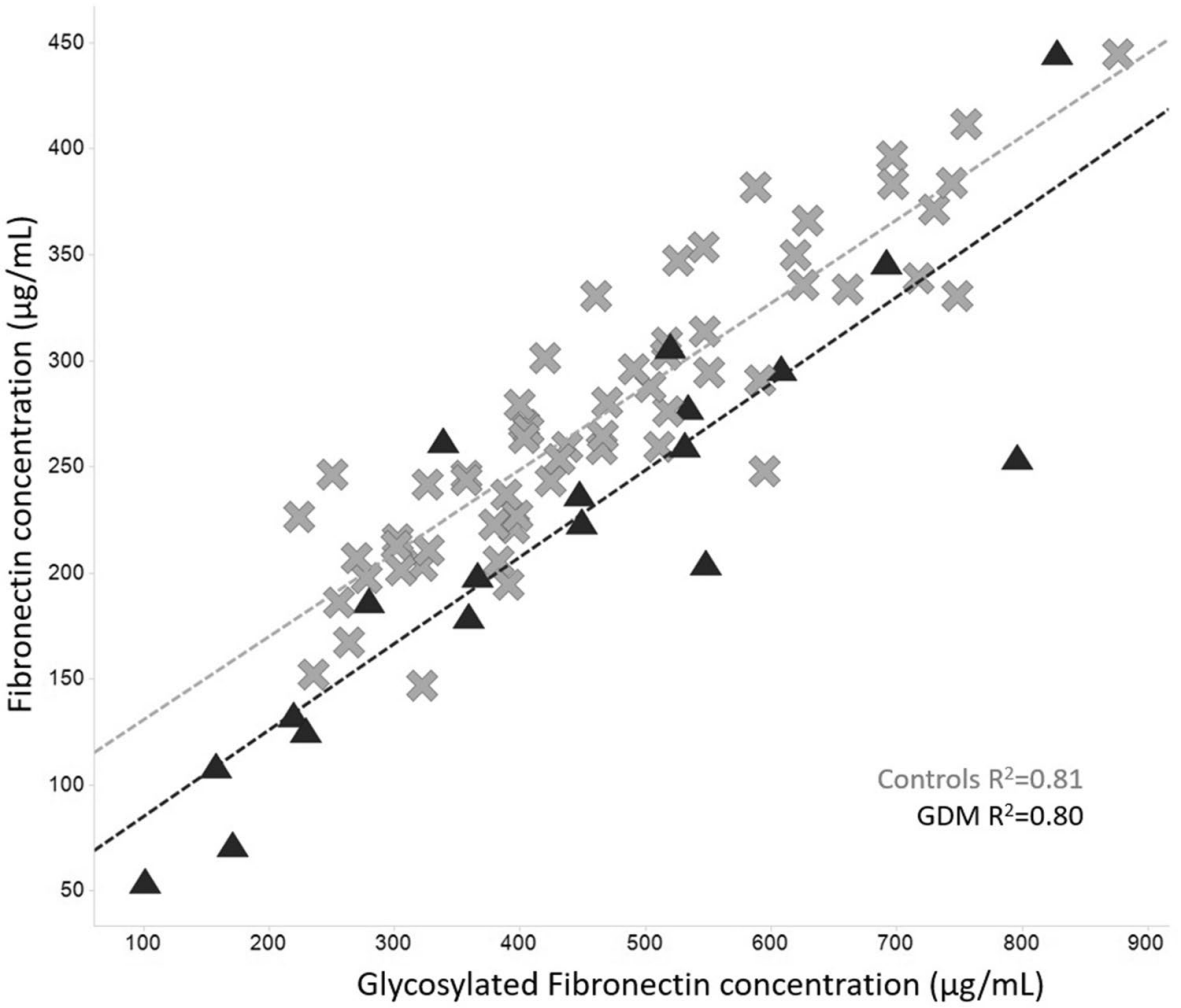
Correlation of glycosylated fibronectin (SNA-based assay) and normal fibronectin (antibody-based assay). There was a high and significant correlation between the assays across control (*n* = 59) and GDM (*n* = 19) groups

OGTT results in GDM and control groups are presented in Fig. 1. The difference at each time point (0, 1 and 2 h) was statistically significant (*p* < 0.01 or *p* < 0.001) between the two groups. Figure 2 shows that there was a high correlation (*R*^2^ ~ 0.8) between glycosylated fibronectin (SNA-based assay) and normal fibronectin (antibody-based assay). Indicating that glycosylation detected by SNA-lectin is not totally independent from normal glycosylation pattern when measuring protein through immunogenic epitopes. Correlation between adiponectin and SNA-reactive adiponectin was low (*R*^2^ < 0.1) in both, GDM and control groups.

**Fig. 3:**
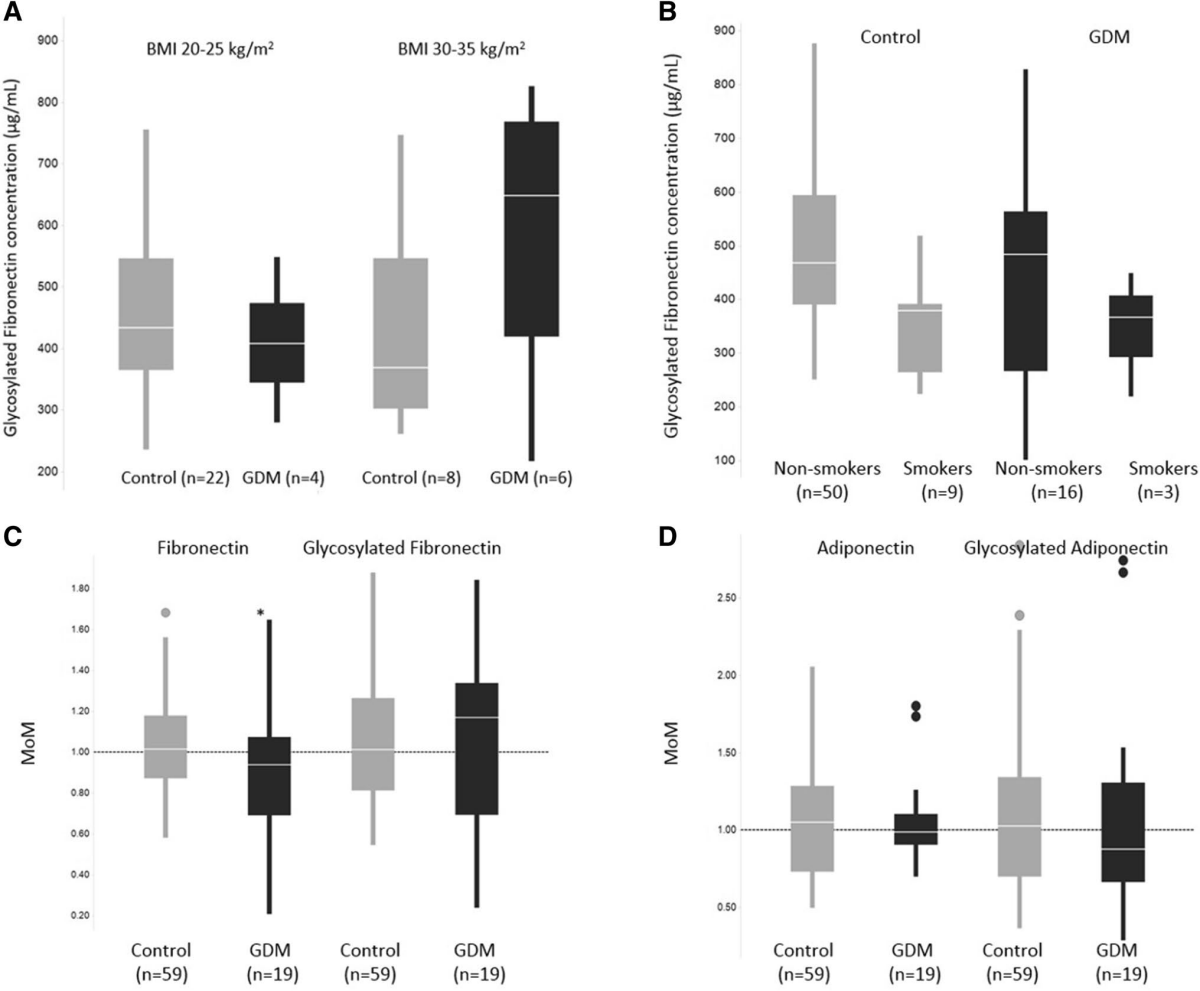
**a** Glycosylated fibronectin concentrations in BMI subgroups of 20–25 and 30–35 kg/m^2^ in the study and the control group. **b** Effect of smoking on concentrations of glycosylated fibronectin in the control and GDM groups. **c** Multiple of median (MoM) values for fibronectin and glycosylated fibronectin between control and GDM groups. **d** Multiple of median (MoM) values for adiponectin and glycosylated adiponectin between control and GDM groups

The difference in glycosylated fibronectin concentrations between the GDM and control women did not differ statistically significantly in different BMI subgroups. In the normal BMI group of 20–25 kg/m^2^, the glycosylated fibronectin levels were 433.9 μg/mL (IQR 365.4–546.6, *n* = 22) in the control group and 407.6 μg/mL (IQR 344.7–473.6, *n* = 4) in the GDM group. In the obese BMI group of 30–35 kg/m^2^, the concentrations were 371.2 μg/mL (IQR 304.3–548.2, *n* = 8) in the control group and 649.9 μg/mL (IQR 421.2–770.1, *n* = 6) in the GDM group (Fig. 3a). Smoking did not alter the glycosylated fibronectin levels statistically significantly between the control and GDM groups: the levels for smokers in these groups were 379.3 μg/mL (IQR 263.8–391.0) and 366.4 μg/mL (IQR 293.0–407.6), respectively, and for the non-smokers in the same control and GDM groups, 467.5 μg/mL (IQR 390.2–594.0) and 483.6 μg/mL (IQR 266.9–563.0), respectively (Fig. 3b).

**Fig. 4:**
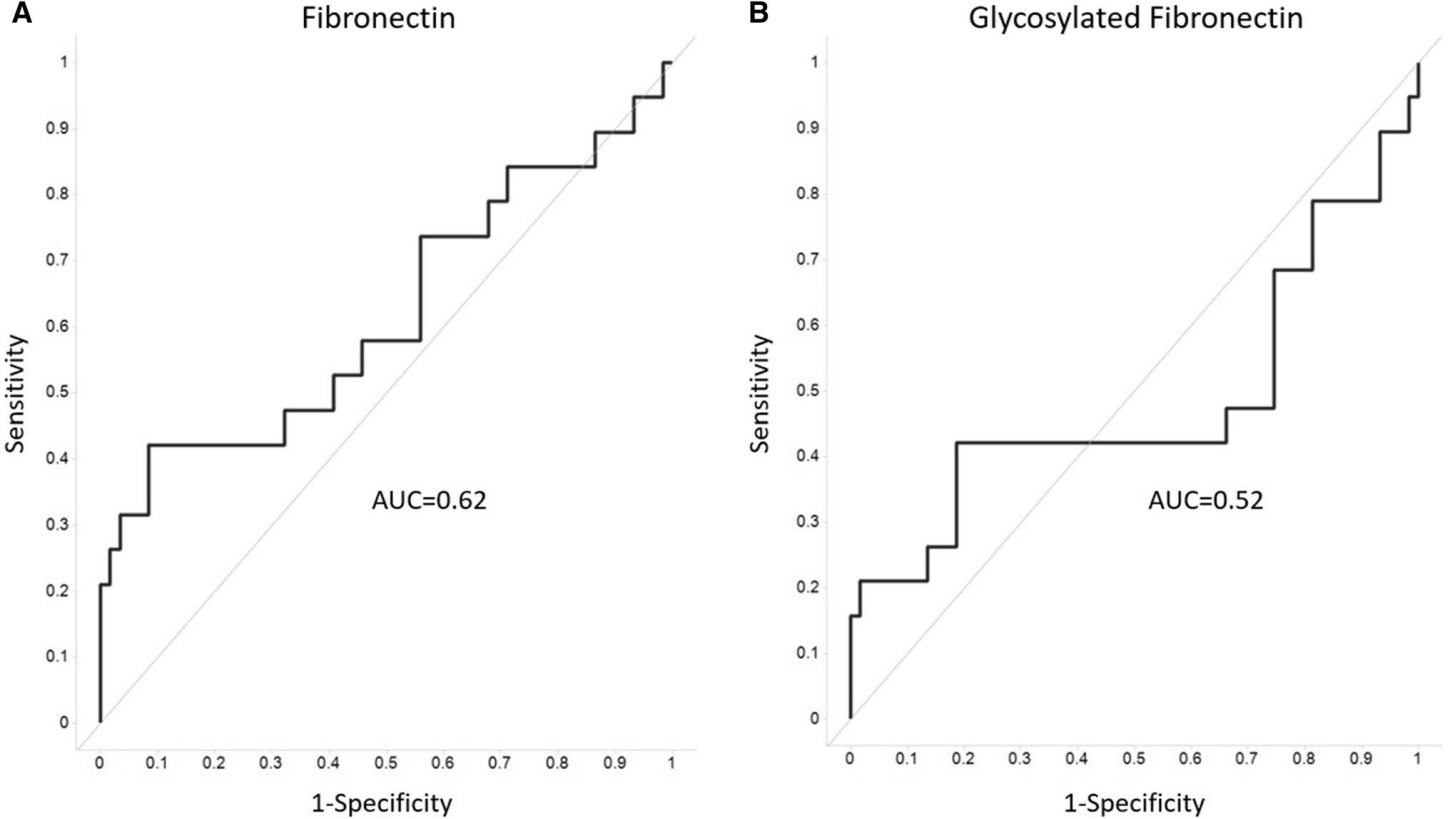
Screening performance using the logistic regression model for fibronectin (**a**) and glycosylated fibronectin (**b**). *AUC* area under the curve

Analyte concentrations were normalized to multiple of median (MoM) values so the possible effect from underlying co-factors, such as gestational age, maternal age, weight, smoking status, could be eliminated. Use of MoM values are widely used in prenatal risk screening, e.g., for aneuploidies and pre-eclampsia. MoM values of fibronectin (*p* = 0.02) and glycosylated fibronectin (*p* = 0.61) as well as adiponectin and glycosylated adiponectin between controls and GDM women are presented in Fig. 3c, d. Logistic regression model was used to evaluate the screening performance of both glycosylated fibronectin and fibronectin for GDM (Fig. 4). With the same sensitivity of 42.1%, fibronectin had better specificity than glycosylated fibronectin, 91.5 and 81.5%, respectively. In the BMI group of 30–35 kg/m^2^, glycosylated fibronectin had a better screening performance: for a sensitivity of 67% the specificity was 67%.

Median adiponectin concentration levels were 141 μg/mL for control samples and 123 μg/mL for GDM samples and were approximately 40% lower than concentrations measured using SNA-lectin assay version, 201 and 173 μg/mL, respectively. Multiple of median (MoM) values of adiponectin and glycosylated adiponectin between controls and GDM women are presented in Fig. 7. Logistic regression model was not evaluated for the screening performance as neither of the adiponectin assays did not show any significance on predicting GDM in this sample set.

## Discussion

In this study, there was no statistical difference in glycosylated fibronectin concentrations in the first trimester maternal serum samples between the GDM group and control group. Fibronectin concentrations were significantly lower in the GDM group compared to controls. BMI and smoking affected glycosylated fibronectin levels. Smokers tended to have lower glycosylated fibronectin concentrations compared to non-smokers and the difference was seen within both GDM and control group. Increase in BMI enhanced glycosylated fibronectin concentration in GDM group but not in the control group. These differences between BMI and smoking subgroups, however, did not reach statistical significance within the present design and statistical assumptions.

Novel screening markers for GDM are needed as currently GDM is mostly diagnosed only in the second trimester of the pregnancy. In Europe, there is a lack of consensus regarding GDM screening and practices and policies vary even within countries [23, 24]. The early detection and treatment for GDM has benefits for both mother and the offspring [25–27]. There is no clear threshold in maternal glucose levels after which risk of pregnancy complications start to enhance but the increase is continuous [28]. Inadequacies in current screening have led to the search of maternal serum markers by which an earlier and more efficient detection of GDM could be reached.

The purpose of this study was to evaluate the screening performance of first trimester maternal serum glycosylated fibronectin as a marker for GDM. Rasanen et al*.* (2013) found in their study a significant difference in glycosylated fibronectin concentrations between the GDM group (132 ± 36 mg/L, *n* = 90) and the control group (80 ± 4.0 mg/L, *n* = 92, *p* < 0.001) [6]. In this study, there was no statistically significant difference between the groups, although glycosylated fibronectin concentrations were slightly higher in the GDM group compared to controls. There was a statistically non-significant increase in the GDM group compared to control group in women with BMI of 30–35 kg/m^2^ suggesting that glycosylated fibronectin might be a better marker in obese women. However, the number of obese women was too small; six women with GDM and eight control women.

Rasanen et al*.* (2013) used a fibronectin monoclonal antibody (MAB1918) as primary antibody in enzyme-linked immunoassay using a Konelab 60i Clinical Chemistry Analyzer. They also used biotin-conjugated *Sambucus nigra* lectin (Vector Labs) in the process. In this study, the same primary coating antibody was pre-treated with Remove iT Endo S to reduce unspecific binding to glycosylated fibronectin. Different laboratory techniques might explain differences in the results. All forms of fibronectin are glycosylated but here the term glycosylated fibronectin refers to specific sialyated glucose that SNA lectin recognizes. Concentration levels from normal pregnancies were not directly comparable to concentration in Rasanen et al*.* (2013) which is due to lack of international standards preparation for calibration, although primary analyte detecting reagents were same.

Nagalla et al*.* (2015) suggested using fibronectin-SNA as a single marker test for GDM in the first trimester of pregnancy. In contrast to results of this study, glycosylated fibronectin (fibronectin-SNA) levels were significantly higher in GDM group (*n* = 15) compared to controls (*n* = 14). There was no difference in maternal pre-pregnancy BMI between the GDM and control groups [13].

Limited number of studies have assessed the role of glycosylated fibronectin in screening for GDM. The published studies are limited in the number of cases as is the current study. There is an ongoing prospective study using maternal serum glycosylated fibronectin and/or OGTT 75 g at 12–15 gestational weeks as a predictor of subsequent GDM [30]. Meanwhile, this study provides information on the screening performance of glycosylated fibronectin in women with GDM. This study also evaluated glycosylated fibronectin levels in different BMI subgroups.

## Conclusions

According to our results, neither first trimester maternal serum glycosylated fibronectin nor serum fibronectin are effective in screening for GDM. Further larger studies in prospective settings are needed to evaluate first trimester glycosylated fibronectin as a screening method for GDM alone and in combination with other markers.

## Abbreviations

BMI: Body mass index
CV: Coefficient of variation
DR: Detection rate
GDM: Gestational diabetes mellitus
FPR: False positive rate
IQR: Interquartile range
MoM: Multiples of the median
OGTT: Oral glucose tolerance test
SHBG: Sex hormone binding globulin
T2DM: Type 2 diabetes mellitus
